# Association of lifestyle and body structure to ocular axial length in Japanese elementary school children

**DOI:** 10.1186/s12886-017-0519-y

**Published:** 2017-07-12

**Authors:** Hiroto Terasaki, Takehiro Yamashita, Naoya Yoshihara, Yuya Kii, Taiji Sakamoto

**Affiliations:** Department of Ophthalmology, Kagoshima University Graduate School of Medical and Dental Sciences, Kagoshima, Japan

**Keywords:** School myopia, Axial length, Lifestyle, Western food

## Abstract

**Background:**

The purpose of this study is to determine whether the lifestyle and body stature are significantly associated with the axial length (AL) of the eyes of Japanese third grade students.

**Methods:**

A prospective, cross sectional, observational study was performed on 122 third grade students consisting of 61 boys and 61 girls ages 8 to 9 years. The AL, body height, body weight, and body mass index (BMI) were measured. The lifestyle was determined by activities such as the daily duration of indoor studying, television viewing, use of computers and smart phones, outdoor activity time, bed time, Japanese or Western dietary habits, and parental myopia were investigated by a questionnaire with three or five grade levels. The relationship between AL and the questionnaire variables were analyzed by Spearman’s correlation analyses.

**Results:**

Westernized dietary habits (*r* = −0.24, *P* = 0.01), duration of computer and smart phone use (*r* = 0.24, *P* = 0.008), parental myopia (*r* = 0.39, *P* < 0.001), body weight (*r* = 0.26, *P* = 0.005), and BMI (*r* = 0.23, *P* = 0.011) were significantly correlated with the AL. Multiple logistic regression analyses showed that the sex [*r* = −0.48; 95% confidence interval (CI) -0.80 to −0.17, *P* = 0.003], body weight (*r* = 0.04; 95% CI 0.02 to 0.07, *P* = 0.038), westernized dietary habits (*r* = −0.30; 95% CI -0.55 to −0.05, *P* = 0.021), and parental myopia (*r* = 0.40; 95% CI 0.20 to 0.61, *P* < 0.001) were significantly and independently correlated with the AL.

**Conclusions:**

The body weight and parental myopia and westernized dietary habits are factors significantly associated with myopia. Changing from Japanese food style to westernized food style might increase the risk of progression of school myopia.

## Background

Myopia is one of the most prevalent disorders of the eye, and very high myopia is a common cause of blindness [1]. The prevalence of myopia has been increasing worldwide, and the socio-economic burden of myopia on the individual and society is considerable [2, 3]. It is becoming a serious problem in Asian countries because the incidence is increasing, and the age of the onset is becoming younger [4–7].

The exact mechanism responsible for the development of myopia has not been determined. While genetics plays a significant role [8], epidemiological studies have shown that the degree of myopia can be affected by the lifestyle and environmental factors such as educational attainment, longer near work time, and shorter outdoor activity time [2, 3]. The difficulty of investigating these issues is because they are cofounding and interacting. Thus, the identification of the true causal factors for myopia is difficult, and it is necessary to collect more information and analyze them more comprehensively.

The results of many cross sectional studies have shown that there is considerable variation in the prevalence of myopia among children of different ethnic backgrounds, different geographic locations, and different ages [3, 9]. Kagoshima is located in the south-eastern part of Japan, and the prevalence of myopia in school children in Kagoshima was determined more than 15 years ago by our department. We found that the prevalence of myopia was low because the children were living in a rural community with less urbanization [10]. This area has now been urbanized but the traditional Japanese lifestyle still remains than in the Tokyo metropolitan area. Thus, it is an ideal area to examine the effects of different lifestyles on the ocular condition of the Japanese school children.

It is well established that a longer axial length (AL) is significantly associated with myopia [11, 12]. More specifically, the AL accounts for a myopic refractive error in some 70% of East Asian children [11–13]. Thus, it would be interesting to investigate the relationship between the AL and the lifestyles of elementary school age children.

The purpose of this study was to determine the relationship between the axial length of the eye and the different body structures and lifestyles of third grade school children.

## Methods

### Ethics statement

All of the procedures used in this study conformed to the tenets of the Declaration of Helsinki, and they were approved by the Ethics Committee of Kagoshima University Hospital. A written informed consent was obtained from all of the subjects and their parents. This study was registered with the University Hospital Medical Network-clinical trials registry (No. UMIN000015239).

### Subjects

This is the initial study of a long term prospective, longitudinal, observational study performed on third grade students in an elementary school of the Faculty of Education of Kagoshima University. The present results included analysis of the body stature, ocular parameters, and questionnaire data at the baseline. Thus, the present results are the cross-sectional results.

A written informed consent was obtained from all of the subjects and their parents. There were 144 students in the third grade including 122 (87.4%) students from whom a parental consent was obtained. The children consisted of 61 boys and 61 girls whose age was 8- or 9-years. The students were examined between November 17, 2014 and December 18, 2014. Color fundus photographs and optical coherence tomographic images taken by 3D OCT-1 Maestro (Topcon, Tokyo, Japan) and AL measured with the OA-2000 Optical Biometer (Tomey, Nagoya, Japan) in students with no known eye diseases were studied. Only the data of the right eyes were statistically analyzed to avoid falsely precise confidence intervals and falsely small *p* values [14].

A questionnaire was answered by the parents. The questionnaire included questions about the average time of daily indoor studying, television viewing, time of computer and smart phone use, duration of outdoor activity, bed time, Japanese or Western style dietary habits, and parental myopia. The dietary habit was graded into 5 levels: Grade 1, totally traditional Japanese style with rice, miso soup, and fish; and Grade 5, totally westernized style with bread, milk, cheese, and meat. The contents of these grades are summarized in Table 1. The body height and weight were obtained from the annual examination records performed in the primary school.

**Table 1:** Contents of questionnaires

Grade	1	2	3	4	5
Indoor studying (hours)	<0.5	0.5 to 1	1 to 2	2 to 3	3<
Television viewing (hours)	<0.5	0.5 to 1	1 to 2	2 to 3	3<
Screen time (hours)	<0.5	0.5 to 1	1 to 2	2 to 3	3<
Outdoor activities (hours)	<0.5	0.5 to 1	1 to 2	2 to 3	3<
Bedtime (time)	Before 21:00	21:00–22:00	22:00–23:00	23:00–24:00	After 24:00
Dietary habit	Western	Moderately Western	Mixed	Modenately Japanese	Japanese
Parental myopia	None	One	Both		

### Statistical analyses

All statistical analyses were performed with the SPSS statistics 21 for Windows (SPSS Inc., IBM, Somers, New York, USA). The significance of the biometric differences between the sexes were calculated by two sample *t* tests. Differences in the answers to the questionnaire between sexes were calculated by Mann-Whitney U tests. The relationship between the AL and the results of questionnaire were analyzed by Spearman’s correlation analyses. Independent associated factors for the AL were determined by multiple logistic regression with a stepwise approach.

## Results

### Biometrics of participants

We studied 122 right eyes of 122 third grade students. The mean AL was 23.39 ± 0.90 mm with a range from 20.52 to 25.80 mm. The mean body height was 132.4 ± 5.5 cm with a range from 115.9 to 146.7 cm. The mean body weight was 28.8 ± 4.8 kg with a range from 18.8 to 41.6 kg. The mean body mass index (BMI) was 16.3 ± 1.9 ranging from 12.5 to 21.4 (Table 2).

**Table 2:** Participant’s data

	Mean ± SD	Range
Boys/Girls	61/61	
Axial length (mm)	23.39 ± 0.90	20.52–25.80
Body height (cm)	132.4 ± 5.5	115.9–146.7
Body weight (kg)	28.8 ± 4.8	18.8–41.6
BMI (kg/m^2^)	16.3 ± 1.9	12.5–21.4

### Results of grading of lifestyle

Because the answer sheets of the questionnaire were collected just after the agreement to participate, the recovery rate of questionnaire was 100% (122 cases). There were several unclassifiable or absence of answers for some questions (see Table 3).

**Table 3:** Results of the questionnaires

Grade	1	2	3	4	5	none
Indoor studying	2 (1.6%)	52 (42.6%)	62 (50.8%)	5 (4.1%)	0 (0%)	1 (0.8%)
Television viewing	14 (11.5%)	42 (34.4%)	44 (36.1%)	17 (13.9%)	2 (1.6%)	3 (2.5%)
Screen time	80 (65.6%)	29 (23.8%)	9 (7.4%)	3 (2.5%)	0 (0%)	1 (0.8%)
Outdoor activities	71 (58.2%)	36 (29.5%)	12 (9.8%)	1 (0.8%)	0 (0%)	2 (1.6%)
Bedtime	11 (9.0%)	81 (66.4%)	28 (23.0%)	0 (0%)	0 (0%)	2 (1.6%)
Dietary habit	1 (0.8%)	37 (30.3%)	64 (52.5%)	17 (13.9%)	1 (0.8%)	2 (1.6%)
Parental myopia	19 (15.6%)	34 (27.9%)	52 (42.6%)			17 (13.9%)

Indoor studying times for most of the students was 1 to 2 h/day; 2 students (1.6%) were placed in Grade 1 (less than half an hour), 52 (42.6%) in Grade 2 (0.5–1 h), 62 (50.8%) in Grade 3 (1–2 h), 5 (4.1%) in Grade 4 (2–3 h), and none in Grade 5 (more than 3 h). One case did not answer.

For television viewing, most children watched television from 30 min to 2 h/day; 14 students (11.5%) were placed in Grade 1 (less than half an hour), 42 (34.4%) in Grade 2 (0.5–1 h), 44 (36.1%) in Grade 3 (1–2 h), 17 (13.9%) in Grade 4 (2–3 h), and 2 (1.6%) in Grade 5 (more than 3 h) for the television viewing times. Three cases (1.6%) did not answer this question.

Most children used computers and smart phones for less than 30 min a day; 80 students (65.6%) were placed in Grade 1 (less than half an hour), 29 (23.8%) in Grade 2 (0.5–1 h), 9 (7.4%) in Grade 3 (1–2 h), 3 (2.5%) in Grade 4 (2–3 h), and none in Grade 5 (more than 3 h). One case did not answer this question (0.8%).

The daily outdoor activity of students was limited; 71 students (58.2%) were placed in Grade 1 (less than half an hour), 36 (29.5%) in Grade 2 (0.5–1 h), 12 (9.8%) in Grade 3 (1–2 h), 1 (0.8%) in Grade 4 (2–3 h), and none in Grade 5 (more than 3 h). Two cases (2.5%) did not answer this question.

Most of the students went to bed between 21:00 to 22:00 h; 11 students (9.0%) were placed in Grade 1 (before 21:00), 81 (66.4%) in Grade 2 (21:00–22:00), 28 (23.0%) in Grade 3 (22:00–23:00), and none in Grade 4 (23:00–24:00) and Grade 5 (after 24:00). Two cases (1.6%) did not answer this question.

For the dietary habits, most student ate a mixture of westernized and traditional Japanese foods but westernized food was dominant; one student (0.8%) was placed in Grade 1, 37 (30.3%) in Grade 2, 64 (52.5%) in Grade 3, 17 (13.9%) in Grade 4, and 1 (0.8%) in Grade 5. Two cases (1.6%) did not answer.

For parental myopia, 19 students (15.6%) were placed in Grade 1 (neither is myopic), 34 (27.9%) in Grade 2 (one is myopic), and 52 (42.6%) in Grade 3 (both are myopic). Seventeen cases (13.9%) did not answer.

### Relationship between AL and lifestyle

**Fig. 1:**
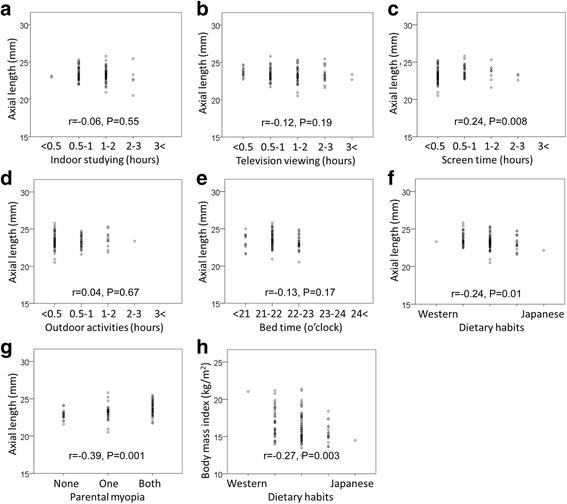
Relationship between axial length (AL) and lifestyle. The hours of the indoor studying (*r* = −0.06, *P* = 0.55), television viewing (*r* = −0.12, *P* = 0.19), outdoor activities (*r* = 0.04, *P* = 0.67), and bedtime (*r* = −0.13, *P* = 0.17) were not significantly correlated with the AL **a**, **b**, **d**, **e**. The duration of computer and smartphone use (*r* = 0.24, *P* = 0.008), westernized dietary habits (*r* = −0.24, *P* = 0.01), and parental myopia (*r* = 0.39, *P <* 0.001) were significantly correlated with the AL **c**, **f**, **g**. Only westernized dietary habits was significantly correlatated with the BMI (*r* = −0.27, *P* = 0.003) **h** although other factors were not

The correlations between the AL and hours of the indoor studying (*r* = −0.06, *P* = 0.55), television viewing (*r* = −0.12, *P* = 0.19), outdoor activities (*r* = 0.04, *P* = 0.67), and bedtime (*r* = −0.13, *P* = 0.17) were not significant (Fig. 1a, b, d, e, respectively). However, the duration time of computer and smartphone use (*r* = 0.24, *P* = 0.008), westernized dietary habits (*r* = −0.24, *P* = 0.01), and parental myopia (*r* = 0.39, *P* < 0.001) were significantly correlated with the AL (Fig. 1c, f, g, respectively).

### Relationship between AL and body stature

**Fig. 2:**
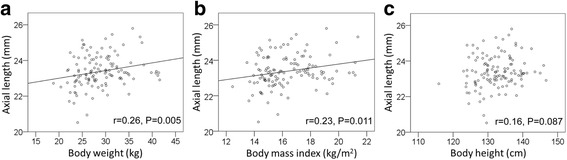
Relationship between AL and body stature. The body weight (*r* = 0.26, *P* = 0.005, **a**) and BMI (*r* = 0.23, *P* = 0.011, **b**) were significantly correlated with the AL but the body height was not (*r* = 0.16, *P* = 0.087, **c**)

The body weight (*r* = 0.26, *P* = 0.005) and BMI (*r* = 0.23, *P* = 0.011) were significantly correlated with the AL but the body height was not (*r* = 0.16, *P* = 0.087; Fig. 2a-c).

### Relationship between BMI and westernized food

Because westernized food was correlated with the AL (Fig. 1f), we examined whether there was a significant correlation between a westernized food diet and BMI which is reportedly involved in the food intake habit [15]. Our results showed that a diet of only westernized food was significantly correlated with the BMI (*r* = −0.27, *P* = 0.003; Fig. 1h). However, other factors were not significantly correlated with the BMI.

### Multiple logistic regression analyses

Multiple logistic regression analyses showed that the independently associated factors for the AL were the male sex [*r* = −0.48; 95% confidence interval (CI) -0.80 to −0.17, standardized coefficient (SC) = −0.26, *P* = 0.003], body weight (*r* = 0.04; 95% CI 0.02 to 0.07, SC = 0.19, *P* = 0.038), westernized dietary habits (*r* = −0.30; 95% CI -0.55 to −0.05, SC = −0.21, *P* = 0.021), and parental myopia (*r* = 0.40; 95% CI 0.20 to 0.61, SC = 0.33, *P* < 0.001).

### Differences between sexes

Because the multivariate analysis showed that the AL was significantly longer in boys than in girls, the differences between the sexes were analyzed in more detail (Table 4). The results showed that there was no significant difference in the body height (131.5 cm vs 133.4 cm, *P* = 0.07), body weight (28.7 kg vs 29.0 kg, *P* = 0.68), and BMI (16.5 vs 16.2, *P* = 0.43) between the boys and girls. The AL was significantly longer in boys than in girls (23.6 vs 23.1 mm, *P* = 0.002). From the answers to the questionnaires, boys spent longer times with a computer and smartphone than girls (*P* < 0.001) and had longer outdoor activity than girls (*P* = 0.015). There was no significant difference in the other questions between the sexes.

**Table 4:** Differences between sexes

	Boys	Girls	*P* value
	*n* = 61	*n* = 61	
Axial length (mm)	23.6 ± 0.9	23.1 ± 0.9	0.002
Body height (cm)	131.5 ± 5.1	133.4 ± 5.7	0.07
Body weight (kg)	28.7 ± 4.5	29.0 ± 5.2	0.68
BMI (kg/m^2^)	16.5 ± 1.8	16.2 ± 2.0	0.43
Indoor studying	2.6 ± 0.6	2.6 ± 0.6	0.991
Television viewing	2.6 ± 1.0	2.6 ± 0.8	0.900
Screen time	1.7 ± 0.8	1.2 ± 0.6	<0.001
Outdoor activities	1.7 ± 0.8	1.4 ± 0.5	0.015
Bed time	2.1 ± 0.6	2.2 ± 0.5	0.700
Dietary habit	2.8 ± 0.7	2.8 ± 0.7	0.787
Parental myopia	1.3 ± 0.8	1.3 ± 0.8	0.972

## Discussion

Our results showed that westernized dietary habits, male sex, body weight, and parental myopia were significantly correlated with the AL in third grade children in Kagoshima, Japan. Westernized food contains more animal fat and simple carbohydrates than Japanese food [15]. It has been reported that the intake of westernized food increases the serum cholesterol in Japanese-Americans, and higher consumption of westernized food has been reported to be associated with higher BMI and obesity in children [15–18]. The BMI was significantly and positively associated with the degree of westernized food intake in our cohort. In earlier studies, a positive and significant correlation was found between the AL and hyper-cholesterolemia and high BMI [19–22]. Although the exact mechanism causing this significant correlation was not determined, we suggest that because saturated fat is a known antagonist of insulin and contributor to insulin resistance which then leads to higher insulinemia [20]. The higher level of insulinemia causes an elongation of the scleral collagen fibrils by increasing the free insulin-like growth factor [19]. Thus, considering the effect of insulinemia, it would be reasonable that those taking westernized food had longer AL than those taking Japanese food because the serum cholesterol would be lower in those who are eating a Japanese diet [15]. On the other hand, several studies have reported that no significant relationship exists between myopia and westernized dietary habit, and Roy et al. showed a negative relationship between them [11, 13]. The reason for these discrepancies could be due to the study population, number of cohorts, and methods of dietary assessment. The residents in Kogoshima area have a wide variety of daily food intake from the traditional Japanese to the westernized food. This diversity of food may have made the effect of dietary habit evident in this study.

Although near work is a known risk factor for myopia, there are problems in quantifying the amount of near work performed which makes it difficult to determining the true effects [2, 3, 23]. We found a significant correlation between the AL and duration of computer and smartphone use by univariate analysis but the correlation was not significant in the multivariate analysis. The widespread use of smartphones is present worldwide, and its adverse effect on health is anticipated other than its effect on near vision. The blue light from the computer screen has a potentially harmful effect on retinal pigment epithelial cells which might affect the elongation of the AL and the development of pathological myopia [24]. However, it does not necessarily mean that near work such as smartphone use plays a causative role in development of myopia. On the contrary, individuals with myopia can use computers and smartphones more easily than those with hyperopia. Additionally, the positive correlation between intelligence and myopia makes a causative role of near work and AL more obscure [25]. From the present results, there may be some, but not independent, effect on the use of computers and smartphones on the AL.

The male sex was an independent and significant factor for a longer AL. In earlier studies of Japanese children, the AL was significantly longer in boys than girls [26, 27] which is consistent with the present data. This may be a characteristic of Japanese children. For the systemic factors, there was no significant difference between boys and girls in body height, body weight, or BMI. On the contrary, the girls were taller than boys even though the difference was not statistically significant (*P* = 0.06). Therefore, this difference was not mainly due to the size of body but other factors. The duration of use of computers and smartphones was significantly longer in boys than girls, and its effect is still unclear.

There have been some recent reports that outdoor activity slows the progression of myopia [28, 29]. Our results showed that outdoor activity was not significantly associated with the AL. In this cohort, 87.7% spent less than 1 h in outdoor activities. This high percentage was probably due to the educational and/or security issues but we were not able to determine the exact reason.

There are limitations in this study. First, the sample size may have been too small to detect slight but significant differences. The fact that some of the variables reached statistical significance indicates that the findings are sound. For example, parental myopia was significantly correlated with the longer AL and boy spent longer times in outdoor activity than girls. These findings are consistent with two other reports [2, 3]. This is understandable because the body structure and the shape of eye is strongly affected by inherited factors.

Second, we examined the AL and not the refractive errors. Even though the AL is longer in myopic eyes, there is no systematic link to myopia [29]. To know the true refractive error of children, it is necessary to use a cycloplegic with parental consent. Unfortunately, an agreement was not always obtained, and so we used the AL without measuring the refractive error. Although an elongation of the AL plays a significant role in the development of myopia in East Asian populations [4, 7–9], this fact should be noted.

Third, our results have a bias because we used a questionnaire method. As a result, factors considered important were not properly quantified, e.g., the absolute degrees of the myopia in the parents will make our analysis more precisely than a simple yes/no answer. This is the limitation of the questionnaire method. Nevertheless, we believe the present results are valid because the well-established factors to be related to myopia such as BMI and parental myopia were confirmed to be significantly correlated.

## Conclusions

We found that a westernized food, male sex, and parental myopia were significantly correlated with the AL in third grade children in Kagoshima city. These findings confirmed that myopia is associated with both environmental and genetic factors [2, 3]. It should be noted that the present study is essentially a cross-sectional study, and the risk of progression of myopia cannot be based on the study findings. At the same time, it is difficult to conclude whether the environmental factors are the cause or the results. Thus, it would be helpful to investigate school myopia in different countries and environments longitudinally to determine which factors are common and causative of myopia.

## Abbreviations

AL: Axial length
BMI: Body mass index
CI: Confidence interal
SC: Standardized coefficient.
